# Double burden of malnutrition among non-pregnant women of reproductive age: Results from the National Health Survey of Panama (ENSPA) 2019

**DOI:** 10.1371/journal.pone.0356784

**Published:** 2026-08-24

**Authors:** Roger Montenegro Mendoza, Flavia Fontes, José Renán De León, Beatriz Gómez, Abdiel Bonilla, Fanny Franco, Bernardo González, Reina Roa, Hedley Quintana, Ilais Moreno Velásquez

**Affiliations:** 1 Department of Research and Health Technology Assessment, Gorgas Memorial Institute for Health Studies, Panama City, Panama; 2 Dietetic and Nutrition Department, University of Panama, Panama City, Panama; 3 Executive Secretariat of the Council of Ministers of Health of Central America, Merliot City, El Salvador; 4 Department of Demography, National Institute of Statistics and Census, Panama City, Panama; 5 Planning Directorate, Ministry of Health, Panama City, Panama; 6 Preventive and Social Medicine Department, University of Panama, Panama City, Panama; Johns Hopkins University Bloomberg School of Public Health, UNITED STATES OF AMERICA

## Abstract

**Background:**

The co-occurrence of different forms of malnutrition has implications for human capital development, as the physiological effects of both anaemia and obesity have been associated with national economic and productivity indicators. This study aims to estimate the prevalence of the double burden of malnutrition including its component conditions, and to identify sociodemographic factors and inflammatory factors associated with the co-occurrence of overweight/obesity and anaemia among non-pregnant women of reproductive age in Panama.

**Methods:**

Using data derived from the 2019 National Health Survey of Panama, we estimated the prevalence of overweight/obesity without anaemia, anaemia without overweight/obesity and the double burden of malnutrition, defined as the co-occurrence of overweight/obesity and anaemia, among non-pregnant women aged 15–49 years. Odds ratios (ORs) with 95% confidence intervals (CIs) were calculated using multinomial logistic regression to assess the association between sociodemographic factors and the double burden of malnutrition.

**Results:**

The weighted prevalence of double burden of malnutrition was 18.6% (95% CI: 15.9%−21.7%). In multivariate adjusted models, compared with women with neither OW/OB nor anaemia, lower vs. higher education (OR= 2.84; 95% CI: 1.24–6.53), ever-having given birth vs. never (OR= 3.01; 95% CI: 1.11–8.15), higher vs. lower inflammation (OR= 9.39; 95% CI: 4.85–18.19) and age 40–49 vs. younger age (OR= 5.99; 95% CI: 1.80–19.95) were associated with double burden of malnutrition. Low education and inflammation were associated with anaemia without overweight/obesity, while older age, indigenous areas, low education, and inflammation were associated with overweight/obesity.

**Conclusion:**

Double burden of malnutrition affected almost one in five women of reproductive age in Panama. While living in indigenous area was associated with overweight/obesity without anaemia, low education and inflammation emerged as potential shared factors for the individual and combined forms of the double burden of malnutrition.

## Introduction

According to the World Health Organization (WHO) estimates, nearly one in three persons is affected by at least one form of malnutrition (undernutrition and or overnutrition) [1]. The double burden of malnutrition (DBM) is defined by the coexistence of undernutrition along with overweight or diet-related non communicable diseases (NCDs), within populations, households and individuals, as well as across the life course [1,2].

At the population level, DBM has been defined as the coexistence, above a defined threshold, of undernutrition (thinness, wasting, underweight, or stunting) and overweight/obesity (OW/OB) among children and/or adults within the same community, country, or geographical region [2]. At the household level, DBM is commonly examined as the coexistence of a child with any form of malnutrition or micronutrient deficiency and a mother who is overweight or obese [3]. At the individual level, DBM is defined as the simultaneous presence of undernutrition indicators, such as stunting, anaemia, or micronutrient deficiencies, and OW/OB in the same individual [2,4,5].

The contributing causes of the DBM relate to a sequence of epidemiological changes known as the nutrition transition, the epidemiological transition, and the demographic transition [4,6]. The accelerating pace at which these three processes have occurred in low- and especially in middle-income countries has resulted in intergenerational changes in diet quality and quantity, leading to a coexistence of undernutrition and overweight within populations, households, and individuals [1,7].

Between 1990 and 2022, the worldwide prevalence of population DBM, defined as the prevalence of underweight/thinness and obesity according to the body mass index (BMI), has increased in most countries. This rise is primarily driven by the rapid increase in obesity rates, which has outpaced the decline in underweight. Moreover, low- and middle-income countries as well as newly high-income countries, have experienced the largest rises in DBM prevalence [8]. Furthermore, it has been hypothesized that, given the nutritional transition diet’s characteristics (energy dense and micronutrient poor), the most prevalent phenotype of DBM is the simultaneous presence of micronutrient malnutrition and OW/OB [5].

The co-occurrence of different forms of malnutrition has implications for human capital development, as the physiological effects of both anaemia and obesity have been associated with national economic and productivity indicators [5,9].

Beyond its health consequences at the individual level, DBM has been associated with economic outcomes, including increased health-care costs and potential productivity losses at the population level.

Furthermore, women’s poor health and malnutrition in all its forms have adverse consequences, increasing the risk of abdominal obesity, hypertension, diabetes, and, consequently, cardiovascular disease in their children, future adults, and ultimately the entire population, thereby perpetuating an intergenerational cycle of malnutrition [5,6,10,11].

Because maternal nutritional status has been associated with long-term health outcomes in both women and offspring, women of reproductive age represent a relevant population in which to examine the prevalence and characteristics of DBM [12].

Although DBM has gained attention in recent years, data on DBM defined as the co-occurrence of micronutrient deficiencies and overnutrition at individual level remain scarce. Furthermore, available reports from Latin American countries were published more than 10 years ago [2,5,13,14].

Panama has been recently reclassified as a high income country in 2024 by the World Bank Group [15], yet it is also considered as one of the most unequal countries in the world, with a Gini index of 49.8 in 2019 widespread poverty, and low quality access to key public services, especially among population living in indigenous territories [16–19].

In Panama, according to the 2019 National Health Survey of Panama, (ENSPA-2019) the prevalence of OW/OB among women aged ≥18 years, was 75.6%. During the same period, the prevalence of anaemia among women of reproductive age was 23.4% [20].

Thus, this study aims to estimate the prevalence of double burden of malnutrition at individual level, describe its components, and identify sociodemographic factors and inflammatory markers associated with the co-occurrence of overweigh/obesity and anaemia among non-pregnant women of reproductive age (NpWRA) in Panama.

## Materials and methods

### Design and sampling

Data were derived from the ENSPA-2019 survey, a nationwide, population-based, cross-sectional study conducted from 1 June to 31 December 2019 to analyse health determinants, environmental, nutritional, and anthropometric factors, as well as access to health services across the Panamanian territory. Eligible participants included individuals of all ages who had resided in their household for at least 6 months and were proficient in the Spanish language.

A complex, randomized, three-stage, stratified cluster sampling method was designed by the National Institute of Statistics and Census to randomly select primary sampling units in its first stage, stratified by area of residence (urban, rural, or indigenous). During the second stage, occupied private homes were chosen at random from the primary sampling units already identified. In the third stage, two individuals were then selected at random from each household: one aged 0 to 14 years and another aged 15 years or older.

The study included the administration of a comprehensive questionnaire, along with anthropometric and blood pressure measurements for all participants. Out of 28,483 participants in ENSPA (including those aged less than 15 years), a total of 17,997 individuals aged ≥15 years were included, representing a weighted population of 3,127,481. For a subsample of participants selected from primary sampling units within the original study, blood sampling and urine tests were also performed. The subsample size was estimated by age group (15–59 years and ≥60 years) based on the proportional demographic distribution of the Panamanian population in these age groups, applied to the originally estimated sample size. The subsample design (n = 5,212) aimed to be representative at national level and by area of residence. More in depth characteristics of the ENSPA-2019 study have been presented elsewhere [20–24].

**Fig 1:**
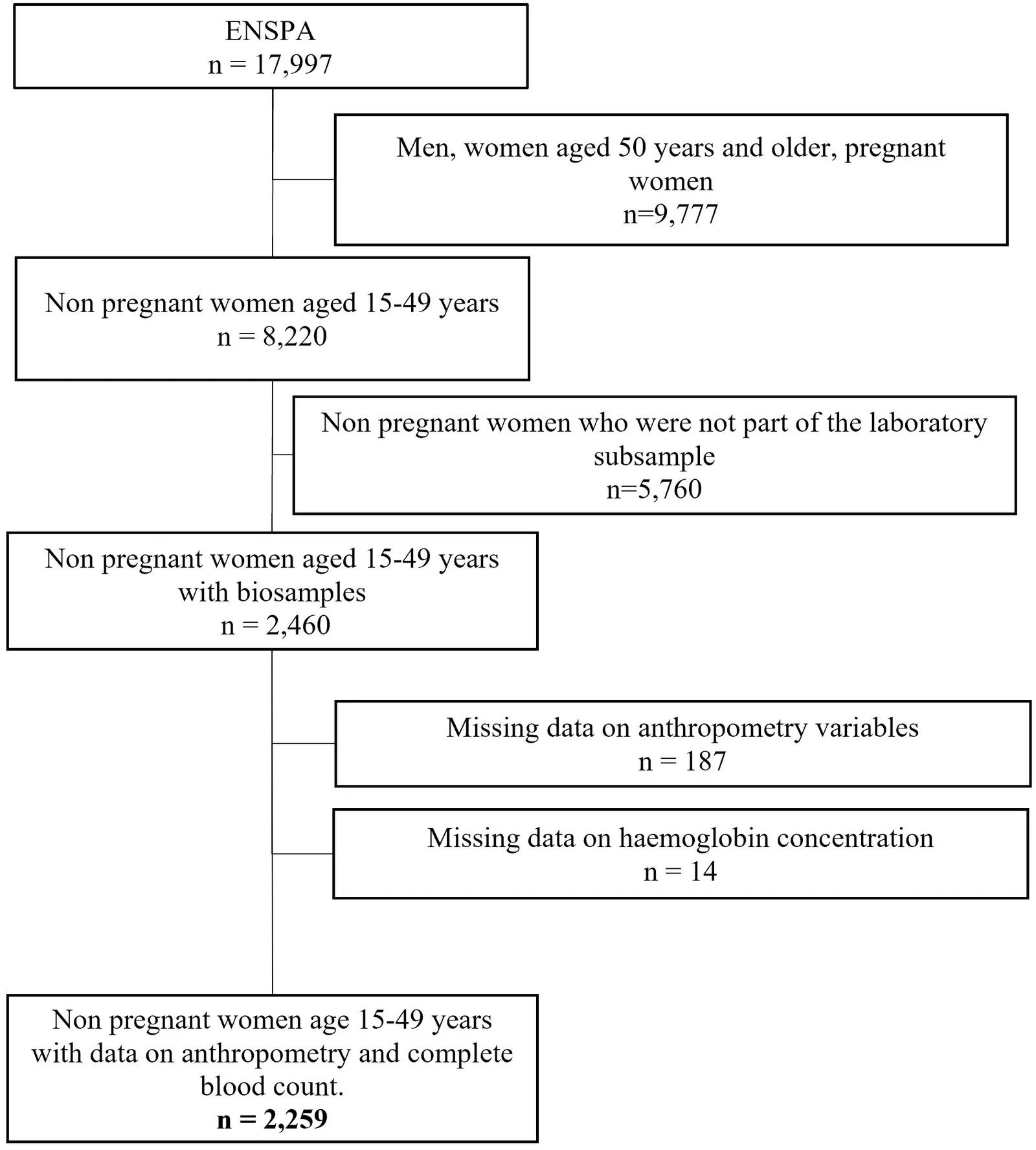
Flowchart summarizing the total ENSPA-2019 participation of individuals aged 15 years and older, and the exclusion and inclusion criteria for the group of non-pregnant women of reproductive age studied in the present analysis.

We restricted our analysis to the group of NpWRA (15–49 years old). Fig 1 shows a flowchart summarizing the total number of participants in the ENSPA-2019 study and the inclusion and exclusion criteria used to determine the subsample of NpWRA age included in the present study. A total of 12,785 individuals did not meet the inclusion criteria for the present analysis because they were not part of the national subsample selected for laboratory analysis, while 2,752 individuals from the subsample were men or women aged >50 years or were pregnant women. Of the remaining participants, additional exclusions were due to missing anthropometric data and haemoglobin values. Thus, in total, n = 2,259 women were included in our analysis.

### Data collection

A wide range of sociodemographic and clinical characteristics were obtained face-to-face through a standardised household questionnaire administered to participants aged ≥15 years, developed for the ENSPA study [20].

All anthropometric measurements were performed in the participant’s household by two standardised trained health personnel. BMI was estimated for all adults participants as the relation of weight in kilograms (kg) divided by the square of the height in metres (m^2^), [25] while for the group age 15–17 years, we determined the Z score for the BMI/age indicator [26].

After completing the questionnaire, participants in the subsample were scheduled for an appointment within the following one to five days. They were instructed to fast for 8–12 hours and to refrain from consuming alcoholic beverages for at least 24 hours prior to sample collection. Blood samples were collected using a standardised operating procedure by trained laboratory technicians.

After collection, samples were processed and conditioned in the laboratories of the provinces where they were obtained, complying with time and cold chain stability specifications. Complete blood counts were conducted within 48 hours after it was collected, as reported elsewhere [20]. Detection limits are presented in the S1 Table.

### Exposures definition

Age was defined as the number of years attained and categorised in 15–19, 20–29, 30–39, and 40–49 years old. Area of residence was defined according to the National Institute of Statistics and Census definitions as urban (localities with at least 1,500 inhabitants with electricity, public water supply, sewage system, paved streets, primary and high schools, commercial establishments, and social and recreational centers); rural (localities with less than 1,500 inhabitants who do not have all or most of the characteristics defined as belonging to the urban area category); and indigenous (all demarcated populated places within the indigenous comarcas).

Ethnic group was self-reported and categorised as Afro-Panamanian, mixed ethnicities, Indigenous, White, Asian or others. Schooling was reported by participants as the highest education level attained and categorised as no schooling or completed elementary school, completed high school, technical or incomplete university, completed university, or others. Labour situation was classified as paid employed, unemployed (and seeking for employment in the last 12 months), or not economically active (neither working nor seeking work in the last 12 months) [27].

Monthly family income was self-reported in USD and categorised in tertiles. Marital status was classified as married or not (which include: single, separated/divorced, and widowed). Ever given birth was determined based on the question, “*Have you ever given birth?*” asked for all women aged ≥15 years.

Ever used tobacco was assessed through the questions: “In your lifetime, how often have you smoked tobacco? and “Have you ever used smokeless tobacco?”. Perceived health status was assessed by asking participants: “Over the last 12 months, how would you describe your state of health?” Responses options were: very good, good, bad or very bad. For the purposes of this study, responses for this variable were dichotomised (very good/good or bad/ very bad).

### Outcomes variables definition

Nutritional status was assessed for participants aged 15−17 years according to the BMI/age indicator, and classified as normal (−2 SD ≤ Z-score ≤ 1 SD), overweight (1 SD < Z-score ≤ 2 SD) or obesity (2 SD < Z-score ≤ 5 SD) [26]. For adults aged ≥18 years, nutritional status was classified according to the BMI as underweight (BMI < 18.5) normal weight (18.5 ≤ BMI < 25), overweight (25 ≤ BMI < 30), or obese (BMI ≥ 30) [25].

Anaemia was defined as a haemoglobin (Hb) concentration below 12.0 g/dL. It was categorised as mild (11.0–11.9 g/dL), moderate (8.0–10.9 g/dL) and severe (<8.0 g/dL). For NpWRA who reported tobacco use in the last 30 days, haemoglobin concentrations were adjusted by subtracting 0.3 g/dL, following WHO guidelines on haemoglobin cut-offs to define anaemia [28].

Anaemia was also classified using the Mean Corpuscular Volume (MCV) and Red Cell Distribution Width (RDW) classification algorithm into: microcytic (MCV < 80 fL), normocytic (MCV: 80–100 fL) or macrocytic (MCV > 100 fL) and increased RDW (≥14.5%) or normal RDW (<14.5%) [29–31]. Inflammation was defined as having a hs-CRP concentration over 5 mg/L [32].

To estimate the prevalence of DBM, we assessed the co-occurrence of OW/OB and anaemia using a four-category dependent variable that classified NpWRA as: neither OW/OB nor anaemia, OW/OB without anaemia, anaemia without OW/OB, and OW/OB with anaemia. DBM was defined as being classified with OW/OB according to the BMI and having anaemia [1,2].

### Statistical analysis

Categorical variables are presented as weighted percentages with their corresponding 95% confidence intervals (CIs). For continuous variables, the weighted median and interquartile range (IQR) are reported. Differences in proportions across categories were explored using the overlap of CIs. The association between OW/OB and anaemia was assessed using a chi-square (χ^2^) test. To analyse the association between the independent variables and the four categories of the dependent variable, we fitted an unconditional multinomial logistic regression model to estimate crude and adjusted odds ratio (ORs) and their 95% CI, using as the reference category the group of women with neither overweight/obesity nor anaemia. The adjusted model included age, area of residence, schooling, and inflammation. Ethnic group, marital status, income, self-perceived health status and tobacco use were excluded as potential confounding variables because of collinearity with other variables or lack of conceptual relevance.

For the hs-CRP, participants with a concentration below the detection limit were assigned the minimum detectable value (eighty-nine participants), and missing values from five participants were imputed using the overall sample median. Similarly, missing values in the schooling (n = 15) and ever given birth (n = 37), were imputed using a simple modal imputation using the mode of participants with the same age and area of residence.

All analyses and results are presented weighted to account for the complex sampling design using the “*svy*” command of the STATA software (version 14; Stata Inc., College Station, TX, USA).

### Statement of ethics

Ethical approval of the ENSPA study was obtained from the Institutional Bioethics Committee of the Gorgas Memorial Institute for Health Studies (749/CBI/ICGES/9 August 2017). All eligible subjects received information about the study prior to participation, and written informed consent was obtained from all participants. For participants aged 15–17 years, written informed assent was obtained, along with written informed consent from their legal guardian.

## Results

Table 1 shows the distribution of NpWRA in weighted frequencies and prevalence according to the exposure variables. From age 20 onwards, the proportion of women was similarly distributed across the ten-year age categories. More than half of the participants lived in urban areas, while the proportion of women from indigenous areas was slightly greater than 5%. Overall, half of the women identified themselves as mixed ethnicity, and more than half had a low education level, were economically inactive, and were married. Two-thirds of women reported a family income below $600.00 and most of the participants had ever given birth, had never used tobacco products, and perceived their health status over the past 12 months as good and very good.

**Table 1:** Distribution of baseline characteristics among non-pregnant women of reproductive age (frequency and weighted prevalence) in the ENSPA study. N = 270,268.

Baseline characteristics	N	Weighted prevalence % (95%CI)
**Ages (in years)**	270,268	
15-19	23,985	8.9 (7.1-10.9)
20-29	81,461	30.1 (27.0-33.4)
30-39	84,099	31.1 (28.1-34.3)
40-49	80,722	29.9 (26.7-33.2)
**Area of residence**	270,268	
Urban	181,869	67.3 (64.9-69.5)
Rural	73,855	27.3 (25.2-29.6)
Indigenous	14,544	5.4 (4.8-5.9)
**Ethnic group**	269,588	
Afro-Panamanian	32,368	12.0 (9.9-14.5)
Mixed ethnicities	136,267	50.5 (47.1-54.0)
Indigenous	37,205	13.8 (11.9-15.9)
White	56,257	20.9 (17.9-24.1)
Asian and others	7,491	2.8 (1.7-4.5)
**Schooling**	269,472	
No schooling or elementary	119,206	44.2 (40.9-47.6)
High school	84,732	31.4 (28.2-34.9)
Technical or incomplete University	30,644	11.4 (9.3-13.7)
University	33,373	12.4 (10.3-14.9)
Other	1,518	0.6 (0.3-1.1)
**Labour situation**	269,588	
Paid employment	56,512	21.0 (18.2-24.0)
Unemployed	48,638	18.0 (15.6-20.8)
Not economically active	164,439	61.0 (57.5-64.3)
**Monthly family income**	255,922	
<$ 250.00	78,039	30.5 (27.7-33.5)
$ 250-599.00	90,906	35.5 (32.1-39.1)
≥ $ 600.00	86,977	34.0 (30.5-37.6)
**Marital status**	269,588	
Married	167,598	62.2 (58.7-65.5)
**Ever given birth**	259,170	
Yes	215,238	83.0 (80.4-85.4)
**Ever used tobacco**	264,265	
Yes	5,284	2.0 (1.3-3.0)
**Perceived health status in the last 12 months**	269,588	
Very good and good	236,858	87.9 (85.4-89.9)
Bad and very bad	32,731	12.1 (10.1-14.6)

Missing values: ethnic group, labour situation, perceived health status and marital status 0.2% (4/2259); monthly family income 5.6% (127/2259), schooling 0.7% (15/2259), ever given birth 1.6% (37/2259), and ever used tobacco 0.2% (5/2259).

S2 Table presents a comparison of women included and excluded from the analysis by age group, area of residence, schooling, and monthly family income. Overall, no statistically significant differences were observed between included and excluded women. Although a statistically significant difference was identified for area of residence, Ethnic group and ever used tobacco, these differences across categories were smaller than 4%.

Table 2 shows the distribution of anthropometric measures, blood biomarkers, nutritional status categories, and the prevalence and severity of anaemia with its classification based on the MCV/ RDW among NpWRA in Panama 2019.

**Table 2:** Distribution of anthropometric variables, blood cell indices and nutritional status among non-pregnant women of reproductive age in the ENSPA study. (N = 270,268).

Anthropometric, and blood cell indices variables		Median (IQR)
Weight (kg)		70.0 (59.3-82.0)
Height (cm)		155.3 (151.0-159.9)
BMI (kg/m^2^)		28.9 (24.7-33.7)
Haemoglobin (g/dl)		12.7 (12.0-13.4)
MCV (fL)		87.4 (83.9-90.6)
RDW (%)		12.6 (12.1-13.4)
hs-CRP (mg/L)*		2.6 (1.0-5.3)
**Nutritional Status**	**Total N = 270,268**	**Weighted prevalence %(95% CI)**
**BMI Categories**		
Underweight (BMI < 18.5 kg/m^2^)%	5,090	1.9 (1.2-2.9)
Normal weight (BMI 18.5–24.9 kg/m^2^) %	64,988	24.1 (21.4-26.9)
Overweight (BMI 25.0–29.9 kg/m^2^) %	81,289	30.1 (27.0-33.3)
Obesity (BMI ≥ 30.0 kg/m^2^) %	118,901	43.9 (40.5-47.5)
**Anaemia (**Hb < 12.0g/dL)	67,038	24.8 (21.8-28.1)
Mild (Hb 11.0–11.9 g/dL)	41,949	15.5 (13.1-18.3)
Moderate (Hb 8.0–10.9 g/dL)	21,836	8.1 (6.3-10.4)
Severe (Hb < 8.0 g/dL)	3,253	1.2 (0.6-2.2)
**Anaemia Classification based on MCV and the RDW**		
Normocytic (MCV 80–100 fL) with normal RDW (≤14.5%)	34,844	12.9 (10.6-15.5)
Microcytic (MCV < 80 fL) with increased RDW (>14.5%)	18,229	6.7 (5.1-8.8)
Microcytic (MCV < 80 fL) with normal RDW (≤14.5%)	7,342	2.7 (1.7-4.3)
Normocytic (MCV 80–100 fL) with increased RDW (>14.5%)	6,412	2.4 (1.5-3.6)
Macrocytic (MCV > 100fL) with increased RDW (>14.5%)	211	0.1 (0.0-0.5)
**Inflammation** (CRP > 5 mg/L)	71,936	26.6 (23.6-29.8)

IQR: Interquartile Range; CI: Confidence Interval; BMI: Body Mass Index; MCV: Mean Corpuscular Volume; fL: Femtoliters; RDW: Red Cell Distribution Width; hs-CRP: High-sensitive C Reactive Protein; *a total of 89 participants had hs-CRP concentrations below the detection limit and were therefore assigned the minimum detectable value of 0.2 mg/L. Additionally, five women (0.2% of the total NpWRA) had missing values for this biomarker, for which a simple imputation using the overall sample median was applied.

Based on BMI categories, 75.9% of NpWRA exhibited malnutrition, of which the majority was due to overweight or obesity 74.0% (95% CI: 71.0%−76.8%), while a small proportion to underweight. Inflammation was estimated in 26.6% and anaemia in 24.8%. Overall, based on haemoglobin concentration, the most frequent form of anaemia was categorised as mild, while based on MCV and the RDW, the most frequent classification was normocytic with normal RDW.

In addition to the total number of NpWRA with anaemia presented in Table 2 there were 2.7% (7,255/270,268) of NpWRA with an increased RDW without anaemia.

The weighted prevalence of women with anaemia who declared to have suffered in the last 12 months any accident or haematological condition that could affect the dynamics of the blood count, such as: amputation, wounds/injuries, major blunt-force injuries, collision, animal attacks, or bruises, was less than 1% (data presented in S3 Table).

**Fig 2:**
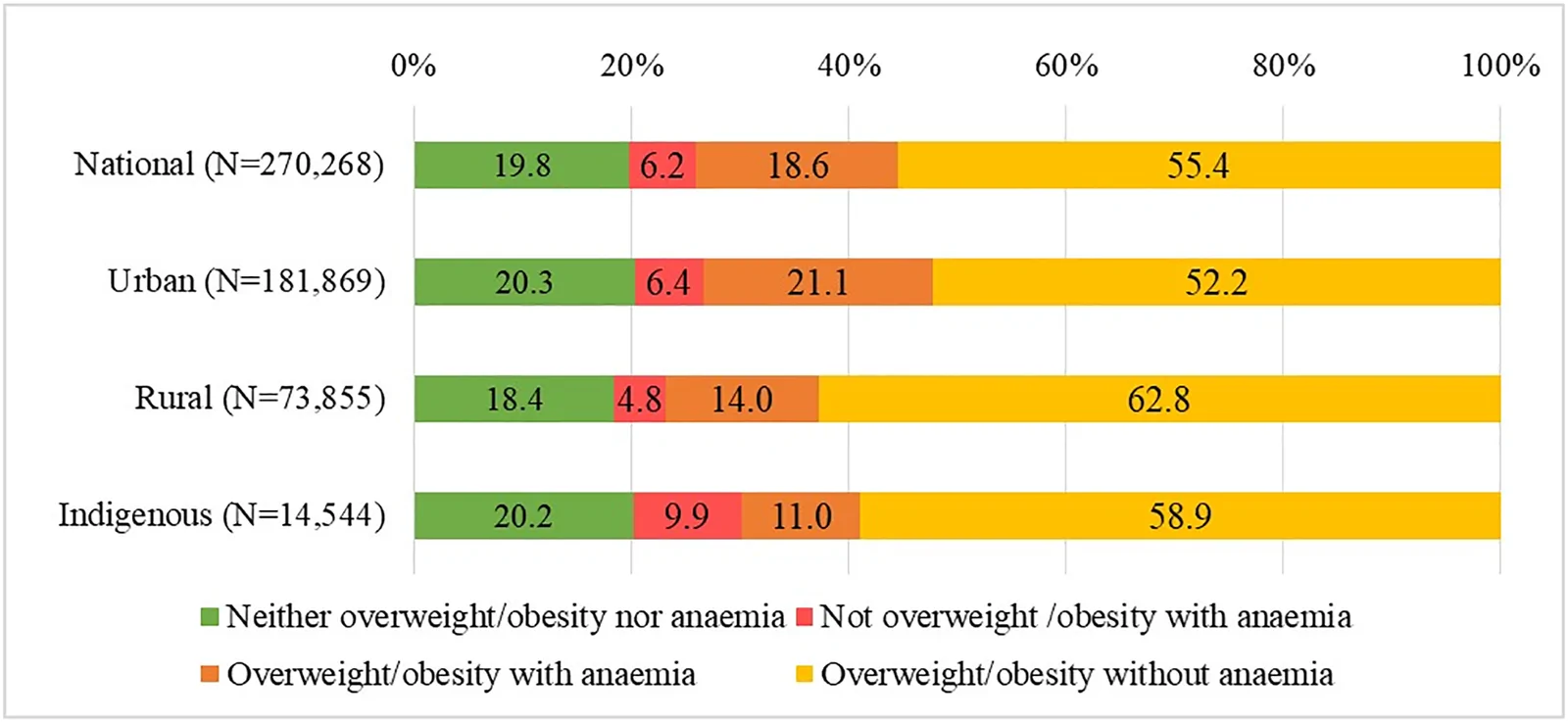
Percentage distribution of non-pregnant women of reproductive age according to the co-occurrence of overweight/obesity and anaemia by area of residence in the ENSPA study (N = 270,268).

Fig 2 depicts the distribution of NpWRA according to the co-occurrence of OW/OB and anaemia by area of residence. At national level the prevalence of NpWRA with DBM was 18.6% (95% CI: 15.9%−21.7%). More than half of the women (55.4%; 95% CI: 52.0%−58.8%) had OW/OB without anaemia, while 6.2% (95% CI: 4.8%−7.9%) had anaemia without OW/OB.

At national level, and consistently across the three areas of residence, one in five NpWRA were neither OW/OB nor anaemic, indicating that approximately 80% experienced at least one form of malnutrition or DBM. The type of malnutrition, however, varied by area: the highest prevalence of DBM was observed in urban areas, the highest proportion of OW/OB without anaemia in rural areas, and the highest prevalence of anaemia without OW/OB in indigenous areas. Although the “Neither OW/OB nor anaemia” category included women with a BMI < 18.5 kg/m^2^, the lowest BMI in our study group was 17.6 kg/m^2^ (S1 Table).

Table 3 presents the distribution of baseline characteristics according to the presence of neither overweight/obesity nor anaemia, overweight/obesity without anaemia, anaemia without overweight/obesity, and double burden of malnutrition.

**Table 3:** Frequency and prevalence of overweight/obesity, anaemia and the co-occurrence of overweight/obesity and anaemia among non-pregnant women of reproductive age according to baseline characteristics in the ENSPA study. (N = 270,268).

Baseline characteristics	N	Weighted prevalence % (95%CI)			
		Neither overweight/obesity nor anaemia	Overweight/obesity without anaemia	Anaemia without overweight/obesity	Overweight/obesity with anaemia
**Total**	270,268	19.8 (17.3-22.5)	55.4 (52.0-58.9)	6.2 (4.8-7.9)	18.6 (15.9-21.7)
**Ages (in years)**	270,268				
15-19	23,985	50.1 (39.4-60.8)	31.2 (22.3-41.7)	8.2 (3.6-17.3)	10.5 (5.1-20.5)
20-29	81,461	23.7 (18.9-29.2)	56.9 (50.6-63.0)	6.6 (4.4-9.8)	12.7 (8.9-17.9)
30-39	84,099	15.8 (12.2-20.3)	57.6 (51.6-63.4)	9.1 (6.1-13.4)	17.5 (13.0-23.1)
40-49	80,722	10.8 (7.6-15.2)	58.9 (52.1-65.3)	2.1 (1.1-3.9)	28.2 (22.4-34.9)
**Area of residence**	270,268				
Urban	181,869	20.3 (17.0-24.0)	52.2 (47.6-56.8)	6.4 (4.6-8.9)	21.1 (17.5-25.2)
Rural	73,855	18.4 (15.1-22.3)	62.8 (57.3-67.9)	4.8 (2.9-7.7)	14.0 (10.0-19.3)
Indigenous	14,544	20.2 (14.4-27.6)	58.9 (50.7-66.7)	9.9 (6.6-14.6)	11.0 (6.9-17.2)
**Ethnic group**	269,588				
Afro-Panamanian	32,368	14.6 (9.4-22.1)	50.3 (40.1-60.5)	4.7 (2.0-10.6)	30.3 (21.6-40.8)
Mixed ethnicities	136,267	22.1 (18.5-26.2)	54.2 (49.4-58.9)	6.7 (4.7-9.6)	16.9 (13.4-21.2)
Indigenous	37,205	18.5 (13.3-25.3)	58.1 (49.6-66.1)	8.6 (5.1-14.2)	14.8 (9.9-21.5)
White	56,257	18.8 (13.6-25.3)	58.1 (49.6-66.2)	4.5 (2.3-8.5)	18.5 (12.3-27.0)
Asian and others	7,491	14.1 (5.2-33.1)	67.2 (44.3-84.1)	2.7 (0.7-9.7)	15.9 (5.2-39.5)
**Schooling**	269,472				
No schooling or elementary	119,206	19.1 (15.6-23.1)	53.1 (48.1-58.0)	9.0 (6.5-12.3)	18.8 (15.1-23.2)
High school	84,732	16.6 (12.6-21.6)	57.9 (51.1-64.3)	4.5 (2.7-7.5)	20.9 (15.5-27.7)
Technical or incomplete University	30,644	23.2 (16.1-32.3)	55.9 (45.8-65.6)	5.0 (2.1-11.6)	15.9 (9.9-24.5)
University	33,373	25.4 (18.1-34.4)	58.3 (48.4-57.5)	1.8 (0.7-4.3)	14.5 (8.4-24.0)
Other**	1,518	61.1 (29.0-85.8)	38.8 (14.1-70.9)	0.0	0.1 (0.0-0.5)
**Labour situation**	269,589				
Paid employment	56,512	15.6 (10.9-21.9)	54.5 (46.4-62.3)	5.0 (2.4-10.2)	24.9 (18.3-32.4)
Unemployed	48,638	22.4 (16.5-29.8)	47.5 (39.8-55.3)	8.1 (4.8-13.3)	21.9 (15.7-29.9)
Not economically active	164,439	20.5 (17.4-23.9)	58.1 (53.8-62.4)	6.0 (4.4-8.2)	15.4 (12.2-19.1)
**Monthly family income**	255,922				
<$ 250	78,039	20.9 (17.3-25.0)	56.7 (51.4-61.9)	7.8 (5.2-25.0)	14.6 (10.9-19.3)
$ 250-599	90,906	18.4 (13.9-23.8)	53.9 (47.6-60.2)	7.2 (4.8-10.6)	20.5 (15.5-26.7)
≥ $ 600	86,977	18.8 (14.6-23.9)	58.0 (51.4-64.4)	3.1 (1.6-6.1)	20.0 (15.1-26.1)
**Marital status**	269,588				
Not married	101,991	27.6 (22.9-32.7)	49.3 (43.7-54.9)	7.6 (5.3-10.9)	15.3 (11.6-20.5)
Married	167,598	15.1 (12.5-18.1)	59.2 (54.8-63.4)	5.3 (3.7-7.5)	20.4 (16.8-24.5)
**Ever given birth**	259,170				
Never	43,933	38.4 (30.7-46.6)	45.2 (37.4-53.2)	7.3 (4.4-12.0)	9.1 (4.9-16.3)
Yes	215,238	16.6 (14.2-19.4)	56.9 (53.1-60.6)	6.1 (4.5-8.1)	20.3 (17.3-23.8)
**Ever used tobacco**	269,549				
Never	264,265	19.9 (17.5-22.7)	55.3 (51.8-58.8)	6.2 (4.8-8.0)	18.5 (15.7-21.6)
Yes	5,284	10.9 (3.3-30.5)	62.6 (40.8-80.2)*	5.2 (1.3-18.0)	21.3 (8.6-43.9)
**Perceived health status in the last 12 months**	269,588				
Very good and good	236,858	20.9 (18.3-23.9)	55.0 (51.2-58.6)	6.2 (4.7-8.1)	17.9 (15.2-21.2)
Bad and very bad	32,731	11.5 (6.1-20.7)	58.9 (48.9-68.3)	6.2 (3.0-12.2)	23.4 (15.9-32.9)
**Inflammation**	270,268				
Yes	198,331	3.6 (2.2-5.8)	69.1 (62.4-75.2)	3.5 (1.7-7.1)	23.8 (18.2-30.4)
No	71,936	25.6 (22.4-29.1)	50.5 (46.5-54.5)	7.1 (5.4-9.3)	16.8 (13.7-20.3)

The prevalence of OW/OB without anaemia increased with age and was estimated at more than half of NpWRA in all age categories above 19 years. Similarly, prevalence exceeded 50% across most baseline characteristic variables; however, among women who had ever smoked tobacco, the prevalence was greater than 60%.

Across the three categories of area of residence, the highest prevalence of OW/OB without anaemia was observed among women living in rural areas, followed by those in indigenous areas. Differences were also observed among married women and among those who had ever given birth, compared with their respective counterparts (non-overlapping 95% CIs).

The prevalence of anaemia without OW/OB varied between 2.1% for the older group (40–49 years) and 9.1% for the 30–39 years group and decreased across the schooling and monthly family income categories. The estimated prevalence of anaemia without OW/OB was the highest for NpWRA living in indigenous areas (non-overlapping 95% CIs).

Prevalence of OW/OB with anaemia increased with age and was the highest for those in the urban area and for women reporting to have ever given birth. Regarding the ethnic group, the DBM among Afro-Panamanian women was twice that of the indigenous women, while those who reported not been economically active as well as those with the lowest family income presented the lowest prevalence.

Estimates for anaemia without OW/OB were similar for the age groups between 15–39 years old and decreased to the lowest for the elder group.

The distribution of NpWRA according to the co-occurrence of OW/OB with anaemia and anaemia classification based on MCV and RDW is presented in S4 Table. In both groups (anaemia without OW/OB and DBM), the highest proportion of anaemia was classified as normocytic with a normal RDW, followed by microcytic anaemia with an increased RDW.

In the model adjusted for age, area of residence, schooling, ever given birth, and inflammation, compared to the reference category (Neither overweight/obesity nor anaemia), the odds of being overweight or obese without anaemia (OR= 6.17; 95% CI: 3.02–12.58) as well as of having DBM (OR= 5.99; 95% CI: 1.80–19.95), were higher among women in the older group vs the younger group (Table 4). No association was observed with age and anaemia without OW/OB. Similarly, compared to the reference category (Neither overweight/obesity nor anaemia), participants living in indigenous areas had higher odds of having OW/OB without anaemia in comparison with those from urban areas (OR= 1.91; 95% CI: 1.01–3.60).

**Table 4:** Crude and adjusted association between baseline characteristics and overweight/obesity without anaemia, anaemia without overweight/obesity and the co-occurrence of overweight/obesity and anaemia among non-pregnant women of reproductive age in the ENSPA study. (N = 270,268).

Baseline characteristics	Crude OR and 95% CI				Adjusted OR and 95% CI			
	OW/OB without anaemia	Anaemia without OW/OB	OW/OB with anaemia	Neither overweight/ obesity nor anaemia	OW/OB without anaemia	Anaemia without OW/OB	OW/OB with anaemia	Neither overweight/obesity nor anaemia
**Ages (in years)**								
15-19	**Reference**	**Reference**	**Reference**	**Reference**	**Reference**	**Reference**	**Reference**	**Reference**
20-29	3.86 (2.19-6.79)	1.71 (0.63-4.63)	2.56 (1.01-6.49)		2.63 (1.36-5.08)	1.63 (0.35-7.63)	1.30 (0.43-3.95)	
30-39	5.85 (3.30-10.37)	3.51 (1.28-9.62)	5.27 (2.10-13.20)		4.14 (2.14-7.99)	3.61 (0.73-17.86)	2.66 (0.85-8.34)	
40-49	8.73 (4.66-16.34)	1.16 (0.36-3.67)	12.40 (4.89-31.47)		6.17 (3.02-12.58)	1.01 (0.19-5.35)	5.99 (1.80-19.95)	
**Area of residence**								
Urban	**Reference**	**Reference**	**Reference**	**Reference**	**Reference**	**Reference**	**Reference**	**Reference**
Rural	1.32 (0.94-1.85)	0.81 (0.42-1.58)	0.73 (0.44-1.21)		1.38 (0.95-1.99)	0.66 (0.33-1.32)	0.75 (0.43-1.29)	
Indigenous	1.13 (0.69-1.84)	1.53 (0.79-2.98)	0.52 (0.27-1.01)		1.91 (1.01-3.60)	1.62 (0.59-4.39)	1.23 (0.51-2.97)	
**Schooling**								
No schooling or elementary	1.21 (0.72-2.02)	6.67 (2.38-18.71)	1.72 (0.81-3.68)	**Reference**	1.79 (1.05-3.08)	10.71 (3.57-32.12)	2.84 (1.24-6.53)	**Reference**
High school	1.52 (0.87-2.65)	3.84 (1.25-11.77)	2.25 (0.99-5.10)		1.81 (1.01-3.24)	4.68 (1.47-14.86)	2.86 (1.17-7.03)	
Technical or incomplete University	1.05 (0.54-2.02)	3.05 (0.78-11.89)	1.19 (0.47-3.03)		1.47 (0.76-2.84)	4.05 (1.01-16.16)	1.96 (0.71-5.39)	
University	**Reference**	**Reference**	**Reference**		**Reference**	**Reference**	**Reference**	
Other**	0.28 (0.07-1.15)	< 0.00	< 0.00		0.78 (0.12-4.89)	< 0.00	<0.00	
**Ever given birth**								
Never	**Reference**	**Reference**	**Reference**	**Reference**	**Reference**	**Reference**	**Reference**	**Reference**
Yes	2.90 (1.93-4.36)	1.91 (0.97-3.79)	5.12 (2.46-10.66)		1.57 (0.92-2.68)	1.75 (0.50-6.14)	**3.01 (1.11-8.15)**	
**Inflammation**	270,268							
No	**Reference**	**Reference**	**Reference**	**Reference**	**Reference**	**Reference**	**Reference**	**Reference**
Yes	9.78 (5.66-16.89)	3.50 (1.37-8.92)	10.12 (5.39-18.99)		9.72 (5.47-17.26)	3.47 (1.31-9.19)	9.39 (4.85-18.19)	
**Ever used tobacco**								
Never	**Reference**	**Reference**	**Reference**	**Reference**				
Yes	2.06 (0.54-7.82)	1.52 (0.25-9.16)	2.10 (0.46-9.64)					
**Perceived health status in the last 12 months**								
Very good and good	**Reference**	**Reference**	**Reference**	**Reference**				
Bad and very bad	1.95 (0.94-4.07)	1.82 (0.64-5.11)	2.38 (1.04-5.44)					
**Ethnic group**								
White	**Reference**	**Reference**	**Reference**	**Reference**				
Afro-Panamanian	1.11 (0.57-2.18)	1.35 (0.41-4.48)	2.10 (0.93-4.74)					
Mixed ethnicities	0.79 (0.49-1.26)	1.27 (0.55-2.94)	0.77 (0.41-1.47)					
Indigenous	1.01 (0.57-1.79)	1.94 (0.73-5.11)	0.81 (0.37-1.77)					
Asian and others	1.54 (0.45-5.21)	0.81 (0.14-4.53)	1.14 (0.223-5.47)					
**Labour situation**								
Paid employment	**Reference**	**Reference**	**Reference**	**Reference**				
Unemployed	0.61 (0.34-1.09)	1.13 (0.39-3.24)	0.61 (0.29-1.26)					
Not economically active	0.82 (0.51-1.31)	0.92 (0.37-2.31)	0.47 (0.26-0.85)					
**Monthly family income**								
<$ 250	0.88 (0.59-1.31)	2.26 (0.95-5.36)	0.66 (0.38-1.14)	**Reference**				
$ 250-599	0.95 (0.60-1.52)	2.37 (0.97-5.76)	1.05 (0.58-1.90)					
≥ $ 600	**Reference**	**Reference**	**Reference**					
**Marital status**								
Not Married	**Reference**	**Reference**	**Reference**	**Reference**				
Married	2.19 (1.55-3.09)	1.26 (0.70-2.29)	2.39 (1.49-3.85)					

For the adjusted analysis a simple modal imputation was performed for cases with missing values in the schooling (n = 15) and have ever given birth (n = 37) variables, using the mode of participants with the same age and area of residence. OW/OB: Overweight or obesity.

Compared to the reference category, educational attainment was inversely associated with the odds of all three malnutrition outcomes. Women with no schooling or only elementary education had the highest odds of anaemia without overweight/obesity (OR= 10.71; 95% CI: 3.57–32.12), overweight/obesity without anaemia (OR= 1.79; 95% CI: 1.05–3.08), and DBM (OR= 2.84; 95% CI: 1.24–6.53), relative to women with university education.

In addition, compared with the reference category, women who had ever given birth showed higher odds of DBM (OR= 3.01; 95% CI: 1.11–8.15) vs those who had never given birth. Furthermore, women with inflammation had higher odds of being classified into any of the three malnutrition outcomes studied.

## Discussion

The DBM among NpWRA represents a significant public health challenge due to its long-term consequences for both individuals and populations. DBM contributes to increases health-care costs and reduced productivity, which may ultimately hinder economic growth. Addressing this form of malnutrition is therefore essential for achieving the Sustainable Development Goals. In Panama, our findings indicate that among NpWRA, the prevalence of overweight/obesity, anaemia and individual-level DBM was 74.0%, 24.8%, and 18.6%, respectively. Older age and having ever given birth were associated with DBM, age and residence in indigenous areas were associated with overweight/obesity without anaemia; and schooling was associated with all the forms of malnutrition examined in this study.

### OW/OB

The estimated prevalence of overall OW/OB in the present study was higher than the worldwide estimates reported for women aged ≥18 years (41.8%; 95% CI: 41.3%−42.4%) and of women living in the Americas regions (65.5%; 95% CI: 64.6%−66.5%) in 2019. The prevalence of OW/OB among NpWRA in our study was similar to that reported among women aged 20–49 years in Mexico 2018–2019 (74.8%); yet the prevalence of obesity in our study was higher than the estimated in Mexico (35.8%) [33].

OW/OB defined as a BMI over 25 kg/m^2^ has shown to be a strong predictor of overall mortality, while obesity (when the BMI exceeds 30 kg/m^2^), is associated with reduced median survival [34].

Obesity is recognised as a chronic complex disease defined by excessive adiposity presenting a risk to health, and it is also considered a risk factor leading to other NCDs [35–37]. Thus, in the context of our findings the currently elevated estimates of overweight/obesity are alarming, firstly because the prevalence of obesity is even higher than that of overweight, and secondly because, at least in this study group, this excess-energy condition does not protect women in childbearing age from the presence of anaemia. Of note, we did not assess adiposity in the present study, which warrants further investigation, as it is possible that body fat distribution or metabolic alterations may influence the coexistence of overweight/obesity and anaemia in women of reproductive age [38].

### Anaemia

Our estimate of anaemia prevalence among NpWRA was lower than the global level prevalence (28.6%; 95% CI: 26.0%−31.2%), but higher than the estimated for the Americas region (16.2%; 95% CI: 13.2%−19.7%), the Latin America and the Caribbean region (18.1%; 95% CI: 13.9%−23.2%) and for the high-income countries group (15.4%; 95% CI: 12.8%−18.7%), according to the WHO estimates for the same year of our study [39].

According to the WHO classification of anaemia as a public problem [40], the prevalence of anaemia among NpWRA in Panama for 2019 represents a moderate level of public health significance. Furthermore, the distribution of anaemia severity showed that mild anaemia was the most prevalent subtype, followed by moderate anaemia [28]. In contrast to a pooled analysis of population-representative studies, the estimates of anaemia by severity in our study were closer to the estimates for Central and Eastern Europe (mild: 14%; moderate: 6.0% and severe: 0.0%) for 2019 [41].

When analysing the anaemia morphologic characteristics based on the MCV-RDW classification scheme, we estimated that half of NpWRA with anaemia had a normal size erythrocyte with a normal RDW, therefore the aetiology for this type of anaemia could be related to anaemia of chronic inflammation, anaemia of renal disease or hereditary spherocytosis [31].

The other half of NpWRA with anaemia was classified mainly as having microcytic anaemia with an increased RDW (which could be related with iron deficiency or sickle-β thalassemia); in a less but similar proportion between having microcytic anaemia with normal RDW (related to α- or β-thalassemia trait or anaemia of chronic inflammation) and normocytic anaemia with increased RDW (associated with an early iron deficiency or vitamin B9, B12 deficiency, or sickle cell anaemia.); lastly macrocytic anaemia with an increased RDW was estimated in less than 1.0% while macrocytic anaemia with normal RDW was not found in our study group [31]. These results indicate that, in this group of NpWRA, less than half of the anaemia could be related to iron deficiency.

Due to the low-grade chronic inflammation associated with excess adiposity, several studies have investigated the relationship between fat mass, iron homeostasis, and the iron deficiency anaemia [38]. Although, in the present study, we estimated the prevalence of different anaemia classification based on MCV and RDW according to the overweight/obesity status (S4 Table) and observed similar estimates between groups, is important to note that applying the new obesity redefinition proposed by the Lancet Diabetes & Endocrinology Commission may have allowed us to distinguish, within the overweight/obesity category, women with excess body fat mass leading to organ dysfunction from those who do not yet exhibit clinically significant complications but remain at increased risk of developing them [42].

In our study, the prevalence of anaemia without OW/OB was similar across women aged 15–39 years but lower among those aged 40–49 years. This was different to an increasing trend in the prevalence of overall anaemia according to age reported for Mexico and Colombia in 2012 but similar to Liberia [43]. Although the number of deliveries and education have been associated with anaemia among women of reproductive age in low- and middle-income countries [44], in our study we only observed associations to this outcome with low education and high hs-CRP. Of note, women who simultaneously had anaemia and OW/OB were grouped into a separate DBM category. In this DBM category, increasing point estimates were seen with higher age, and had ever given birth was associated with the outcome. Removing these older and high-risk women from the anaemia without OW/OB group likely diluted any age-related association in our result.

Even though monthly family income was not included in the adjusted model, the descriptive analysis showed that the prevalence of anaemia without OW/OB was the lowest among women in the highest family income group. This pattern is consistent with findings from the BRINDA project, which showed that anaemia is less common among populations with greater socioeconomic advantage, regardless of infection burden [43].

### Co-occurrence of OW/OB & Anaemia

The DBM at the individual level have been reported by other authors [13,45–49]. In a study analysing data from nationally representative surveys in 17 different countries worldwide between 2006–2014 including low-, middle- and high-income countries (HIC), the estimated prevalence of OW/OB and anaemia among NpWRA ranged from 1.0% in Vietnam for 2010 to 18.6% in Afghanistan in 2013. While in high-income countries like United States of America and United Kingdom, this estimate was 4.6% and 4.8% respectively [13].

Using Demographic and Health Surveys conducted between 2000 and 2019 in 52 low- and middle-income countries (LMIC), the overall pooled prevalence of the co-occurrence of OW/OB and anaemia was estimated at 12.4% (95% CI: 11.1%−13.7%), among non-pregnant women aged 20−49 years, and more specifically for the Americas region, based on data from Bolivia (2008), Guyana (2009), Honduras (2011), Peru (2012), Guatemala (2014) and Haiti (2016), the estimates were 13.5% (95% CI:10.5%−16.4%) [14,49].

These results indicate that, despite Panama’s recent classification as HIC, the prevalence of DBM exceeds estimates reported for LMIC and the Americas region. This pattern may reflect the country’s current stage of nutritional transition rather than its economic classification alone. According to this framework, Panama may be characterised as being in the stage 4 of the nutritional transition, marked by increased consumption of fat, sugary drinks, and ultra-processed foods [20]; dietary changes affecting even indigenous groups [50]; technological and lifestyles shifts with high levels of physical inactivity (54.9%) [23]; and a high prevalence of obesity and NCD’s [20,51,52].

Consistent with previous studies, [13,53–55] we found a positive association between age and both OW/OB without anaemia and the DBM, possibly reflecting, among others, age-related physiological changes that decrease energy expenditure. Women residing in indigenous areas had higher odds of OW/OB without anaemia compared with urban women, which may be partly explained by the outcome categorisation, as women residing in urban areas with OW/OB were divided between two categories, and a higher proportion was found for the DBM category. Additionally, lower educational attainment was associated with higher odds of overweight/obesity, anaemia, and DBM, and having ever given birth was associated with DBM, potentially reflecting disparities in health literacy, access to health services, health information and life-course demands [56].

Finally, women with inflammation had higher odds of presenting OW/OB, anaemia or DBM compared with women without inflammation and with neither condition, with the strongest association observed for groups including OW/OB. This finding is consistent with the well-documented relationship between excess adiposity and systemic inflammation [57,58], as CRP levels are typically elevated in individuals with overweight and obesity due to the chronic low-grade inflammatory state associated with excess adipose tissue and especially with visceral fat.

Another phenotype of the DBM that has been explored is the co-occurrence of cardiometabolic risk factors with anaemia or micronutrient deficiencies. Although this analysis only examines the prevalence of the co-occurrence of OW/OB and anaemia, additional analysis, including dyslipidaemia, hypertension and diabetes mellitus, should be explored in this population.

## Conclusion

The estimated prevalence of the double burden of malnutrition at individual level, defined as the co-occurrence of overweight/obesity and anaemia among non-pregnant women of reproductive age in Panama for 2019, was 18.6% (95% CI: 15.9%−21.7%).

After adjusting for age group, area of residence, schooling, ever given birth and inflammation, the odds of DBM increased with age and were higher among women who had ever given birth compared with those who had not. Additionally, while living in indigenous area was associated with overweight/obesity without anaemia, low education and inflammation emerged as potential shared factors for the individual and combined forms of the double burden of malnutrition.

Addressing the double burden of malnutrition among non-pregnant women of reproductive age in Panama is essential to end all forms of malnutrition and to achieve the Sustainable Development Goals. Our findings highlight the importance of a socioeconomic gradient across the malnutrition spectrum. From a public health perspective, the implementation of multicomponent interventions aimed at reducing economic inequalities, fostering healthy food environment, and ensuring nutritional adequacy, particularly among vulnerable population groups, may help reduce the health and social impacts associated with all forms of malnutrition.

### Limitation

Due to the cross-sectional design of this study, causal relationship between the variables cannot be inferred. The presence of unmeasured confounders or mediators cannot be excluded.

Although we use an algorithm scheme classification to describe the type of anaemia, is important to underscore that this classification is only suggestive of the potential causes but not an absolute classification. Thus, there might be an overlap of the RDW values among some of the conditions in each MCV category. Additionally, the MCV represents a mean value that could appear as normal in the early stage of a disease or when multiple aetiologies coexist in the same person [30,31,59,60]. However, this was beyond the scope of this study. Finally, after applying the inclusion and exclusion criteria for the present analysis, the final sample size of NpWRA was smaller than the total ENSPA sample, raising the possibility of selection bias and potentially affecting the representativeness of the findings. Nevertheless, comparisons between women included and excluded from the analysis showed no statistically significant differences in age, education level, or family income. Although a statistically significant difference was observed for area of residence, the magnitude of this difference was small.

### Strengths

ENSPA is the largest cross-sectional recent study conducted in Panama so far, with a standardised collection of information. Complete blood count used to estimate the prevalence and severity of anaemia for the ENSPA study was performed using venous blood with automated haematology analysers and high-quality control measures as recommended by the WHO guidelines on haemoglobin to define anaemia in individuals and populations [28].

The distribution of NpWRA included in this study, according to age and geographic area, was like that observed for the women of reproductive age at national level, according to the census results in 2023 [61].

## Supporting information

S1 Table Anthropometric variables and biomarkers according to descriptive statistics. BMI: Body Mass Index; MCV: Mean Corpuscular Volume; RDW: Red Cell Distribution Width; IQR: Interquartile Range; SD: Standard deviation; *hs-CRP: High-sensitive C Reactive Protein; a total of 89 participants had hs-CRP concentrations below the detection limit and were therefore assigned the minimum detectable value of 0.2 mg/L, while five women (0.2%) had missing values for this biomarker. N/A: Not Applicable.
(PDF)

S1 Appendix Detailed data collection process and instruments. (PDF)

S2 Table Baseline characteristics of non-pregnant women of reproductive age of the ENSPA-2019 according to inclusion/exclusion criteria from the present study. (PDF)

S3 Table Percentage distribution of non-pregnant women of reproductive age reporting accident or non-cancer haematological conditions in the past 12 months, by presence of anaemia. Panama, 2019.
(PDF)

S4 Table Proportion of non-pregnant women of reproductive age according to the co-occurrence of overweight/obesity with anaemia and anaemia classification based on the Mean Corpuscular Volume and the Red Cell Distribution Width in the ENSPA study. MCV: Mean Corpuscular Volume; RDW: Red Cell Distribution Width; fL: Femtolitres; CI: Confidence Interval.
(PDF)
