# Supplementary material for: Double burden of malnutrition among non-pregnant women of reproductive age: Results from the National Health Survey of Panama (ENSPA) 2019
Source: PLoS One. 2026 Aug 24;21(8):e0356784. doi: 10.1371/journal.pone.0356784 (PMC13502655; doi:10.1371/journal.pone.0356784)
Supplement: Supplementary file 1 [file pone.0356784.s001.pdf]

S1 Table. Anthropometric variables and biomarkers according to descriptive statistics among non-pregnant women of reproductive age in the ENSPA study, 2019. N=270,268.

Anthropometrics variables	N	Minimum	Maximum	Mean	SD	Median	IQR	Skewness	Kurtosis	Measurement range
Weight (kg)	2,259	32.4	151	71.9	17.7	70.0	59.3-82.0	0.86	4.41	0-400
Height (cm)	2,259	100.4	184	155.5	7.3	155.3	151.0-159.9	-0.39	6.94	0-210
BMI (kg/m^2)	2,259	17.6	51.9	28.9	7.0	28.9	24.7-28.9	1.02	5.33	N/A
**Biomarkers**										
Haemoglobin g/dl	2,259	7.9	15.2	12.6	1.3	12.7	12.0-13.4	-1.08	5.59	0.0-25
MCV (fL)	2,259	54.7	108.1	86.5	6.7	87.4	83.9-90.6	-1.23	6.32	58-139
RDW %	2,259	10	25.1	12.9	1.4	12.6	12.1-13.4	1.86	9.8	10-29.8
hs-CRP mg/L*	2,170	0.2	29.9	4.6	6.1	2.7	1.1-5.6	5.3	62.0	0.1-160
