# Supplementary material for: Double burden of malnutrition among non-pregnant women of reproductive age: Results from the National Health Survey of Panama (ENSPA) 2019
Source: PLoS One. 2026 Aug 24;21(8):e0356784. doi: 10.1371/journal.pone.0356784 (PMC13502655; doi:10.1371/journal.pone.0356784)
Supplement: Supplementary file 2 [file pone.0356784.s002.pdf]

S1 Appendix: Detailed data collection process and instruments

Anthropometric measurements:

All anthropometric measurements were performed in the participant's household by two standardized trained health personnel. For each anthropometric variable (weight and height), two measurements were done with an absolute maximal difference accepted between measurements of 0.5 kilograms for weight, and 0.5 centimetres for height. Otherwise, a third measurement was performed, and the average of the two closest measurements was recorded and used for the analysis for each participant.

Weight was measured using a portable digital scale SECA model 874dr with a precision of 0.1 kg, while height was measured to the nearest millimetre with a stadiometer SECA model 213 I equipped with level. BMI was estimated for all adult participants as the relation of weight in kilograms (kg) divided by the square of the height in meters (m2), while for the group age 15-17 years we determined the Z score for the BMI/age indicator.

Blood sampling:

After completing the questionnaire, participants in the subsample were scheduled for an appointment within the following one to five days, depending on the specific logistical constraints of each health region. They were instructed to fast for 8-12 hours and to refrain from consuming alcoholic beverages for at least 24 hours prior to sample collection. On the day of the appointment, transportation to the designated laboratory health centre for blood sampling was provided. Blood samples were collected using a standardized operating procedure by trained laboratory technicians.

Upon arrival at the laboratory, participants completed a brief additional questionnaire to record their fasting status and medication intake. Blood samples were collected using a standardized operating procedure by trained laboratory technicians. The process began with bar-coding participants' names and identification numbers. Following aseptic and antiseptic standards, venepuncture was performed in the antecubital area to obtain blood sample, which was then distributed in EDTA and serum tubes.

After collection, samples were processed and conditioned in the laboratories of the provinces where they were obtained, complying with time and cold chain stability specifications. Then, sample tubes were retrieved, transported and analysed centrally at Panama City by Hospital Nacional (ISO 15189 certified contractor) in containers between 4 and 8 degrees Celsius. A total of 13 biomarkers were analysed. Complete blood counts were conducted within 48 hours after it was collected as reported elsewhere. [20] Complete blood count was measured using the analyser Ruby Abbot, applying the optics and impedance method, and high sensitive C Reactive Protein (hs-CRP) using the immunoturbidimetry methodology.
