# Supplementary material for: Double burden of malnutrition among non-pregnant women of reproductive age: Results from the National Health Survey of Panama (ENSPA) 2019
Source: PLoS One. 2026 Aug 24;21(8):e0356784. doi: 10.1371/journal.pone.0356784 (PMC13502655; doi:10.1371/journal.pone.0356784)
Supplement: Supplementary file 3 [file pone.0356784.s003.pdf]

S2 Table. Baseline characteristics among non-pregnant women of reproductive age in the ENSPA study according to the inclusion or exclusion from the present analysis (frequency and weighted prevalence).

Baseline characteristics	Non pregnant Women of Reproductive Age from the ENSPA-2019						p-value
	Included in the analysis			Excluded from the analysis			
	Sample size	Weighted		Sample size	Weighted		
		N	Prevalence % (95%CI)		N	Prevalence % (95%CI)	
**Ages (in years)**	2,259	270,268	100	5,961	690,205	100	
15-19	214	23,985	8.9 (7.1-10.9)	594	71,489	10.4 (8.9-11.9)	
20-29	706	81,461	30.1 (27.0-33.4)	2,011	231,308	33.5 (31.5-35.5)	0.06
30-39	737	84,099	31.1 (28.1-34.3)	1,938	210,565	30.5 (28.632.4)	
40-49	602	80,722	29.9 (26.7-33.2)	1,418	176,842	25.6 (23.8-27.5)	
**Area of residence**	2,259	270,268	100	5,961	690,205	100	
Urban	1,046	181,869	67.3 (64.9-69.5)	2,324	437,697	63.4 (61.9-64.9)	0.01
Rural	1,006	73,855	27.3 (25.2-29.6)	2,745	202,077	29.3 (27.9-30.7)	
Indigenous	207	14,544	5.4 (4.8-5.9)	892	50,431	7.3 (6.8-7.9)	
**Ethnic group**	2,255	269,588	100	5,946	685,997	100	
Afro-Panamanian	242	32,368	12.0 (9.9-14.5)	765	105,265	15.3 (13.8-17.0)	
Mixed ethnicities	1,118	136,267	50.5 (47.1-54.0)	2,789	343,231	50.3 (47.9-52.1)	
Indigenous	433	37,205	13.8 (11.9-15.9)	1,393	105,185	15.3 (14.0-16.8)	0.03
White	403	56,257	20.9 (17.9-24.1)	871	113,931	16.6 (15.0-18.3)	
Asian and others	59	7,491	2.8 (1.7-4.5)	128	17,664	2.5 (1.9-3.4)	
**Schooling**	2,244	269,472	100	5,926	683,684	100	
No schooling or elementary	1,163	119,206	44.2 (40.9-47.6)	3,287	331,673	48.5 (46.4-50.63)	
Secondary	586	84,732	31.4 (28.2-34.9)	1,431	196,202	28.7 (26.76-30.71)	
Short-cycle tertiary education or incomplete University	236	30,644	11.3 (9.3-13.7)	574	70,925	10.37 (9.17-11.71)	0.30
University	244	33,373	12.4 (10.3-14.9)	592	81,541	11.93 (10.58-13.42)	
Other	15	1,518	0.6 (0.3-1.1)	42	3,343	0.49 (0.29-0.81)	
**Labour situation**	2,255	269,588	100	5,946		100	
Paid employment	339	56,512	21.0 (18.2-24.0)	1,033	168,184	24.5 (22.7-26.5)	
Unemployed	417	48,638	18.0 (15.6-20.8)	909	126,194	18.4 (16.8-20.1)	0.10
Not economically active	1,499	164,439	61.0 (57.5-64.3)	4,004	4391,619	57.1 (54.9-59.2)	
**Monthly family income**	2,132	255,922	100	5,605	683,684	100	
< $ 250.00	984	78,039	30.5 (27.7-33.5)	2,834	222,654	34.2 (32.4-36.12)	0.17
$ 250-599.00	651	90,906	35.5 (32.1-39.1)	1,547	216,263	33.2 (31.13-35.44)	
≥ $ 600.00	497	86,977	34.0 (30.5-37.6)	1,224	211,532	32.5 (30.43-34.68)	
**Marital status**	2,255	269,588	100	5,946	685,997	100	0.73
Married	1,478	167,598	62.2 (58.7-65.5)	4,040	431,222	62.9 (60.8-64.9)	
**Ever given birth**	2,222	259,170	100	5,884	674,507	100	0.72
Yes	1,844	215,238	83.0 (80.4-85.4)	4,995	563,681	83.6 (81.9-85.0)	
**Ever used tobacco**	2,254	264,265	100	5,939	684,870	100	0.01
Yes	52	5,284	2.0 (1.3-3.0)	141	659,326	3.7 (2.9-4.7)	
**Perceived health status in the last 12 months**	2,255	269,588	100	5,946	685,997	100	
Very good and good	1,980	236,858	87.9 (85.4-89.9)	5,382	615,625	89.7 (88.3-90.9)	0.14
Bad and very bad	275	32,731	12.1 (10.1-14.6)	564	70,372	10.3 (9.0-11.7)	

p-value: indicates whether the distribution of each sociodemographic characteristic differed significantly between women included in the analysis and those excluded due to missing data, accounting for the complex survey design.
