# Supplementary material for: Double burden of malnutrition among non-pregnant women of reproductive age: Results from the National Health Survey of Panama (ENSPA) 2019
Source: PLoS One. 2026 Aug 24;21(8):e0356784. doi: 10.1371/journal.pone.0356784 (PMC13502655; doi:10.1371/journal.pone.0356784)
Supplement: Supplementary file 4 [file pone.0356784.s004.pdf]

Supplementary Table 3. Percentage distribution of non-pregnant women of reproductive age reporting accident or non-cancer haematological conditions in the past 12 months, by presence of anaemia. Panama, 2019.

Condition affecting the dynamics of the blood count results in the last 12 months	Presence of anaemia			
	Yes		No	
	N	% CI	N	% CI
**Total**	67,038	100	203,230	100
**Yes***	140	0.2 (0.0-1.4)	2,638	1.3 (0.7-2.3)
**No**	66,898	99.8 (97.7-99.3)	200,592	98.7% (97.7-99.3)

*Contusion, severe blows or bruises; fracture, sprain or dislocation; wound; laceration; amputation; crushing; multiple wounds; organ damage or impairment, falls, poisoning or intoxication; collision; being run over; snake bite; haematological disease other than cancer.
