# Supplementary material for: Double burden of malnutrition among non-pregnant women of reproductive age: Results from the National Health Survey of Panama (ENSPA) 2019
Source: PLoS One. 2026 Aug 24;21(8):e0356784. doi: 10.1371/journal.pone.0356784 (PMC13502655; doi:10.1371/journal.pone.0356784)
Supplement: Supplementary file 5 [file pone.0356784.s005.pdf]

S4 Table. Proportion of non-pregnant women of reproductive age according to the co-occurrence of overweight/obesity with anaemia and anaemia classification based on the Mean Corpuscular Volume and the Red Cell Distribution Width in the ENSPA study.

Anaemia classification based on MCV and RDW	Co-occurrence of overweight/obesity with anaemia			
	Anaemia without overweight/obesity		Overweight/Obesity with anaemia	
	N	Weighted prevalence % (95 % CI)	N	Weighted prevalence % (95 % CI)
	**16,679**	**100.0**	**50,359**	**100.0**
Microcytic (MCV<80fL) with RDW ≤ 14.5%	1,101	6.6 (2.2-18.3)	6,240	12.4 (7.4-19.9)
Microcytic (MCV<80fL) with RDW > 14.5%	3,720	22.3 (13.4-34.6)	14,510	28.8 (21.6-37.3)
Normocytic (MCV 80 100 fL) with RDW ≤ 14.5%	10,146	60.8 (47.8-72.5)	24,698	49.0 (40.3-57.8)
Normocytic (MCV 80 100 fL) with RDW > 14.5%	1,501	9.0 (4.3-18.0)	4,911	9.7 (5.8-15.9
Macrocytic (MCV > 100 fL) with RDW > 14.5%	211	1.3 (0.2-8.6)	0	0.0
