# Supplementary material for: Double burden of malnutrition among non-pregnant women of reproductive age: Results from the National Health Survey of Panama (ENSPA) 2019
Source: PLoS One. 2026 Aug 24;21(8):e0356784. doi: 10.1371/journal.pone.0356784 (PMC13502655; doi:10.1371/journal.pone.0356784)
Supplement: Peer review history

# Peer review: Decision Letter 0

**DOI:** 10.1371/journal.pone.0356784.r001

**Authors:** Guoying Wang

7 May 2026

Dear Dr. Montenegro Mendoza,

Please submit your revised manuscript by Jun 21 2026 11:59PM. If you will need more time than this to complete your revisions, please reply to this message or contact the journal office at plosone@plos.org. When you're ready to submit your revision, log on to https://www.editorialmanager.com/pone/ and select the 'Submissions Needing Revision' folder to locate your manuscript file.

- A letter that responds to each point raised by the academic editor and reviewer(s). You should upload this letter as a separate file labeled 'Response to Reviewers'.
- A marked-up copy of your manuscript that highlights changes made to the original version. You should upload this as a separate file labeled 'Revised Manuscript with Track Changes'.
- An unmarked version of your revised paper without tracked changes. You should upload this as a separate file labeled 'Manuscript'.

If you would like to make changes to your financial disclosure, please include your updated statement in your cover letter. Guidelines for resubmitting your figure files are available below the reviewer comments at the end of this letter.

If applicable, we recommend that you deposit your laboratory protocols in protocols.io to enhance the reproducibility of your results. Protocols.io assigns your protocol its own identifier (DOI) so that it can be cited independently in the future. For instructions see: https://journals.plos.org/plosone/s/submission-guidelines#loc-laboratory-protocols. Additionally, PLOS ONE offers an option for publishing peer-reviewed Lab Protocol articles, which describe protocols hosted on protocols.io. Read more information on sharing protocols at https://plos.org/protocols?utm_medium=editorial-email&utm_source=authorletters&utm_campaign=protocols.

As the corresponding author, your ORCID iD is verified in the submission system and will appear in the published article. PLOS supports the use of ORCID, and we encourage all coauthors to register for an ORCID iD and use it as well. Please encourage your coauthors to verify their ORCID iD within the submission system before final acceptance, as unverified ORCID iDs will not appear in the published article. *Only*  the individual author can complete the verification step; PLOS staff *cannot*  verify ORCID iDs on behalf of authors.

We look forward to receiving your revised manuscript.

Kind regards,

Guoying Wang, MD, PhD

Academic Editor

PLOS One

Journal Requirements:

When submitting your revision, we need you to address these additional requirements.

1. Please ensure that your manuscript meets PLOS ONE's style requirements, including those for file naming. The PLOS ONE style templates can be found at

https://journals.plos.org/plosone/s/file?id=wjVg/PLOSOne_formatting_sample_main_body.pdf and

https://journals.plos.org/plosone/s/file?id=ba62/PLOSOne_formatting_sample_title_authors_affiliations.pdf

2. We note that the grant information you provided in the ‘Funding Information’ and ‘Financial Disclosure’ sections do not match.

When you resubmit, please ensure that you provide the correct grant numbers for the awards you received for your study in the ‘Funding Information’ section.

3. Thank you for stating the following financial disclosure: [The ENSPA study was funded by the Ministry of Health of Panama via Inter-American Development Bank. HQ and IMV are affiliated with the National Research System (“SNI” in Spanish).].

Please state what role the funders took in the study.  If the funders had no role, please state: ""The funders had no role in study design, data collection and analysis, decision to publish, or preparation of the manuscript.""

If this statement is not correct you must amend it as needed.

Please include this amended Role of Funder statement in your cover letter; we will change the online submission form on your behalf.

4. We note that you have included the phrase “data not shown” in your manuscript. Unfortunately, this does not meet our data sharing requirements. PLOS does not permit references to inaccessible data. We require that authors provide all relevant data within the paper, Supporting Information files, or in an acceptable, public repository. Please add a citation to support this phrase or upload the data that corresponds with these findings to a stable repository (such as Figshare or Dryad) and provide and URLs, DOIs, or accession numbers that may be used to access these data. Or, if the data are not a core part of the research being presented in your study, we ask that you remove the phrase that refers to these data.

5. Thank you for stating the following in the Acknowledgments Section of your manuscript: [The author thanks all the ENSPA-2019 participants, as well as the staff who participated in the data collection process. HQ and IMV are affiliated with the National Research System (“SNI” in Spanish), which is funded by the National Secretary of Science and Technology (“SENACYT” in Spanish).]

We note that you have provided funding information that is not currently declared in your Funding Statement. However, funding information should not appear in the Acknowledgments section or other areas of your manuscript. We will only publish funding information present in the Funding Statement section of the online submission form.

Please remove any funding-related text from the manuscript and let us know how you would like to update your Funding Statement. Currently, your Funding Statement reads as follows: [The ENSPA study was funded by the Ministry of Health of Panama via Inter-American Development Bank. HQ and IMV are affiliated with the National Research System (“SNI” in Spanish).].

Please include your amended statements within your cover letter; we will change the online submission form on your behalf.

6. If the reviewer comments include a recommendation to cite specific previously published works, please review and evaluate these publications to determine whether they are relevant and should be cited. There is no requirement to cite these works unless the editor has indicated otherwise.

[Note: HTML markup is below. Please do not edit.]

Reviewers' comments:

Reviewer's Responses to Questions

**Comments to the Author**

1. Is the manuscript technically sound, and do the data support the conclusions?

Reviewer #1: Yes

Reviewer #2: Yes

Reviewer #3: Yes

**********

2. Has the statistical analysis been performed appropriately and rigorously?

Reviewer #1: Yes

Reviewer #2: Yes

Reviewer #3: Yes

**********

3. Have the authors made all data underlying the findings in their manuscript fully available?

The PLOS Data policy

Reviewer #1: Yes

Reviewer #2: Yes

Reviewer #3: Yes

**********

4. Is the manuscript presented in an intelligible fashion and written in standard English?

Reviewer #1: Yes

Reviewer #2: Yes

Reviewer #3: Yes

**********

Reviewer #1: The topic is extremely important. With increasing burden of non communicable diseases , research focusing on the risk factors is the need of hour.

The pandemic of obesity is alarming and being obese does not ensure that the individual has no nutritional deficiencies which is supported by evidence in this article.

The article is well written

Reviewer #2: This manuscript examines the double burden of malnutrition among non-pregnant women of reproductive age in Panama using nationally representative data. The topic is very relevant to current public health concerns, and I appreciate the use of a large dataset and appropriate statistical approach. Overall, the manuscript is well organized, and the findings are clearly presented and generally supported by the data.

However, the final analytical sample is smaller than the original survey sample due to exclusions. A short discussion on possible selection bias and whether the final sample remains representative would be useful.

Overall, I found this to be a solid and relevant study that adds to the literature on the double burden of malnutrition. With a few clarifications and minor revisions, it would be suitable for publication.

Reviewer #3: The introduction provides a strong rationale for the study and clearly highlights the public health importance of the double burden of malnutrition. However, the tone at times leans toward a normative or prescriptive perspective. I suggest reframing these sections to use a more analytical and descriptive tone, focusing on the epidemiological transitions and drivers rather than on societal deficits.

For example, on page 1, the manuscript states:

“Malnutrition in all its forms impacts the Human Capital Index and, therefore, the sustainable growth and poverty reductions of nations.”

While this is a valid point of view, it is a normative framing of the problem. To shift toward a more positive or analytical academic style, this could be reframed to focus on the measurable relationship between health variables, such as:

“The co-occurrence of different forms of malnutrition represents a challenge for human capital development, as the physiological impacts of both anemia and obesity have been shown to correlate with economic and productivity indicators at the national level.”

Adopting this descriptive approach throughout the introduction will align the background more closely with the objective, data-driven nature of the results and the rigorous multinomial analysis you have performed.

The authors state that the laboratory subsample (n = 5,212) was designed to be nationally representative and representative by area of residence. However, given the logistical requirements for biosample collection there is a potential risk of selection bias or non-participation bias compared to the full ENSPA-2019 survey sample (n = 17,997).

To strengthen the validity of the findings regarding the double burden of malnutrition, it would be beneficial for the authors to include a comparative analysis of the sociodemographic characteristics (e.g., age, education level, wealth quintile, and geographic region) between the full survey sample and the laboratory subsample of NpWRA.

Specifically:

Table Comparison: Could the authors provide a table (as an appendix or within the results) comparing these key characteristics to demonstrate that no significant systematic differences exist?

Bias Discussion: If differences are found, please discuss how these might impact the prevalence estimates and how the complex sampling weights used in the analysis account for this potential bias.

This comparison would provide greater confidence that the subsample effectively preserves the representativeness of the original survey. If found that there is an important difference in distribution weights might need to be recalcualted.

The data collection section is very comprehensive and provides a clear understanding of the study's execution. However, as the authors note that this methodology has been described in detail elsewhere (e.g., in the primary ENSPA-2019 report or previous publications), the current description in the manuscript is quite lengthy.

To improve the flow and readability of the publication, I suggest the following:

Summarize the Core Methods: Keep only the essential information necessary for the reader to understand the current analysis (e.g., the specific variables used for the double burden of malnutrition and the laboratory subsample flow).

Move Detailed Protocols to an Annex: Transfer the more granular procedural details—such as specific equipment brands, detailed field staff training protocols, and step-by-step biosample handling—to a Supplementary Materials or Annex section.

Cross-Reference: Ensure that the text clearly cites the original methodological publication or the new Annex for readers who require the full technical details.

This adjustment would allow for a more concise manuscript that focuses more directly on the study’s specific findings regarding non-pregnant women of reproductive age.

The Discussion section (pages 31–40) provides a thorough comparison of the study’s findings with similar data from other countries. However, much of the current text is dedicated to repeating the results and noting that they are "consistent with" or "similar to" findings in other regions. To increase the impact of the paper, I suggest the authors reduce the repetitive description of results and instead focus on the underlying reasons for these similarities or differences.

Specifically, the manuscript would benefit from a deeper analysis of the "Panamanian paradox." For example:

Economic Classification vs. Health Outcomes: Panama is classified as a High-Income Country (HIC), yet your results for the double burden of malnutrition and anemia are strikingly similar to those found in Low-Income Countries (LIC) or lower-middle-income regions.

Drivers of Disparity: Rather than just stating that the results match other countries, please expand on why this occurs in Panama. Is it due to the specific nature of the country's high income inequality (as mentioned with the Gini index), the rapid pace of the nutrition transition, or specific gaps in the food system that transcend national wealth?

Shifting the focus from what is happening in other countries to why Panama mirrors certain socioeconomic contexts despite its HIC status would provide a much more robust and insightful discussion.

**********

what does this mean?). If published, this will include your full peer review and any attached files.

If you choose “no”, your identity will remain anonymous but your review may still be made public.

**Do you want your identity to be public for this peer review?**  For information about this choice, including consent withdrawal, please see our Privacy Policy

Reviewer #1: No

Reviewer #2: **Yes:** Saadia Asiedu

Reviewer #3: No

**********

[NOTE: If reviewer comments were submitted as an attachment file, they will be attached to this email and accessible via the submission site. Please log into your account, locate the manuscript record, and check for the action link "View Attachments". If this link does not appear, there are no attachment files.]

To ensure your figures meet our technical requirements, please review our figure guidelines: https://journals.plos.org/plosone/s/figures

You may also use PLOS’s free figure tool, NAAS, to help you prepare publication quality figures: https://journals.plos.org/plosone/s/figures#loc-tools-for-figure-preparation.

NAAS will assess whether your figures meet our technical requirements by comparing each figure against our figure specifications.

# Author comment: Author response to Decision Letter 1

**DOI:** 10.1371/journal.pone.0356784.r002

15 Jun 2026

Journal Requirements:

When submitting your revision, we need you to address these additional requirements.

1. Please ensure that your manuscript meets PLOS ONE's style requirements, including those for file naming. The PLOS ONE style templates can be found at

https://journals.plos.org/plosone/s/file?id=wjVg/PLOSOne_formatting_sample_main_body.pdf and

https://journals.plos.org/plosone/s/file?id=ba62/PLOSOne_formatting_sample_title_authors_affiliations.pdf

Response: The file names were revised and adjusted as indicated in the guidelines.

2. We note that the grant information you provided in the ‘Funding Information’ and ‘Financial Disclosure’ sections do not match.

When you resubmit, please ensure that you provide the correct grant numbers for the awards you received for your study in the ‘Funding Information’ section.

Response: Thank you for pointing this mismatch. The authors would like to clarify that the ENSPA study was funded by the Ministry of Health of Panama through a loan from the Inter-American Development Bank, and no grant number was associated with this funding. In addition, the authors did not receive any specific funding for the preparation of this manuscript.

3. Thank you for stating the following financial disclosure: [The ENSPA study was funded by the Ministry of Health of Panama via Inter-American Development Bank. HQ and IMV are affiliated with the National Research System (“SNI” in Spanish).].

Please state what role the funders took in the study. If the funders had no role, please state: ""The funders had no role in study design, data collection and analysis, decision to publish, or preparation of the manuscript.""

If this statement is not correct you must amend it as needed.

Please include this amended Role of Funder statement in your cover letter; we will change the online submission form on your behalf.

Response: The statement was eliminated from the manuscript and included in the cover letter as follows:

“The ENSPA study was funded by the Ministry of Health of Panama through a loan from the Inter-American Development Bank, and no grant number was associated with this funding. HQ and IMV are affiliated with the National Research System ("SNI" in Spanish), which is funded by the National Secretariat of Science, Technology and Innovation ("SENACYT" in Spanish). The funders had no role in the study design, data collection and analysis, decision to publish, or preparation of the manuscript.”

4. We note that you have included the phrase “data not shown” in your manuscript. Unfortunately, this does not meet our data sharing requirements. PLOS does not permit references to inaccessible data. We require that authors provide all relevant data within the paper, Supporting Information files, or in an acceptable, public repository. Please add a citation to support this phrase or upload the data that corresponds with these findings to a stable repository (such as Figshare or Dryad) and provide and URLs, DOIs, or accession numbers that may be used to access these data. Or, if the data are not a core part of the research being presented in your study, we ask that you remove the phrase that refers to these data.

Response: The table presenting the information was added as Supplementary table 3 and rewrote the phrase “data not shown” as “data presented in S3 Table”.

5. Thank you for stating the following in the Acknowledgments Section of your manuscript: [The author thanks all the ENSPA-2019 participants, as well as the staff who participated in the data collection process. HQ and IMV are affiliated with the National Research System (“SNI” in Spanish), which is funded by the National Secretary of Science and Technology (“SENACYT” in Spanish).]

We note that you have provided funding information that is not currently declared in your Funding Statement. However, funding information should not appear in the Acknowledgments section or other areas of your manuscript. We will only publish funding information present in the Funding Statement section of the online submission form.

Please remove any funding-related text from the manuscript and let us know how you would like to update your Funding Statement. Currently, your Funding Statement reads as follows: [The ENSPA study was funded by the Ministry of Health of Panama via Inter-American Development Bank. HQ and IMV are affiliated with the National Research System (“SNI” in Spanish).].

Response: Funding-related information was removed from the revised manuscript.

Please include your amended statements within your cover letter; we will change the online submission form on your behalf.

Response: Thank you very much. The funding statement was amended as follow: “The ENSPA study was funded by the Ministry of Health of Panama via Inter-American Development Bank. HQ and IMV are affiliated with the National Research System (“SNI” in Spanish) which is funded by the National Secretary of Science and Technology (“SENACYT” in Spanish).” And added to the cover letter as recommended.

6. If the reviewer comments include a recommendation to cite specific previously published works, please review and evaluate these publications to determine whether they are relevant and should be cited. There is no requirement to cite these works unless the editor has indicated otherwise.

Review Comments to the Author

Reviewer #1:

1. The topic is extremely important. With increasing burden of non communicable diseases , research focusing on the risk factors is the need of hour.

The pandemic of obesity is alarming and being obese does not ensure that the individual has no nutritional deficiencies which is supported by evidence in this article.

The article is well written

Response: The authors thank the Reviewer #1 for the comment.

Reviewer #2

1. This manuscript examines the double burden of malnutrition among non-pregnant women of reproductive age in Panama using nationally representative data. The topic is very relevant to current public health concerns, and I appreciate the use of a large dataset and appropriate statistical approach. Overall, the manuscript is well organized, and the findings are clearly presented and generally supported by the data.

However, the final analytical sample is smaller than the original survey sample due to exclusions. A short discussion on possible selection bias and whether the final sample remains representative would be useful.

Overall, I found this to be a solid and relevant study that adds to the literature on the double burden of malnutrition. With a few clarifications and minor revisions, it would be suitable for publication.

Response: Thank you for raising this important point. We acknowledge the concern regarding the potential selection bias arising from the limited sample size of NpWRA included in our analysis. To address this concern, we originally presented in Supplementary table #2 the baseline characteristics of NpWRA from the laboratory subsample included in this study and those from the same laboratory subsample who were excluded due to missing anthropometric/haemoglobin data. Nevertheless, to provide a more comprehensive assessment of the potential selection bias, we revised the Supplementary table #2 and now present comparisons (with their respective p-value for their chi2 test) by age group, area of residence, schooling, and family income, between the study sample and the total population of NpWRA from the ENSPA study.

Therefore, we added to the results section the following paragraph to lines 285–293 of the manuscript with track changes:

“S2 Table presents a comparison of women included and excluded from the analysis by age group, area of residence, schooling, and monthly family income. Overall, no statistically significant differences were observed between included and excluded women. Although a statistically significant difference was identified for area of residence, Ethnic group and ever used tobacco, this differences across categories were smaller than 4%.

Additionally, we discuss the comparison between included and excluded women in the limitation section, (lines 577-583) as follows:

“Finally, after applying the inclusion and exclusion criteria for the present analysis, the final sample size of NpWRA was smaller than the total ENSPA sample, raising the possibility of selection bias and potentially affecting the representativeness of the findings. Nevertheless, comparisons between women included and excluded from the analysis showed no statistically significant differences in age, education level, or family income. Although a statistically significant difference was observed for area of residence, the magnitude of this difference was small.”

Reviewer #3

1. The introduction provides a strong rationale for the study and clearly highlights the public health importance of the double burden of malnutrition. However, the tone at times leans toward a normative or prescriptive perspective. I suggest reframing these sections to use a more analytical and descriptive tone, focusing on the epidemiological transitions and drivers rather than on societal deficits.

For example, on page 1, the manuscript states:

“Malnutrition in all its forms impacts the Human Capital Index and, therefore, the sustainable growth and poverty reductions of nations.”

While this is a valid point of view, it is a normative framing of the problem. To shift toward a more positive or analytical academic style, this could be reframed to focus on the measurable relationship between health variables, such as:

“The co-occurrence of different forms of malnutrition represents a challenge for human capital development, as the physiological impacts of both anemia and obesity have been shown to correlate with economic and productivity indicators at the national level.”

Adopting this descriptive approach throughout the introduction will align the background more closely with the objective, data-driven nature of the results and the rigorous multinomial analysis you have performed.

Response: The authors thank for this observation and recommended paragraph. We agree with the reviewer and modify the indicated paragraph. We also reviewed the introduction section to adopt a descriptive approach as follows:

Lines 84-89:

“The co-occurrence of different forms of malnutrition has implications for human capital development, as the physiological effects of both anaemia and obesity have been associated with national economic and productivity indicators. [9] [5]

Beyond its health consequences at the individual level, DBM has been associated with economic outcomes, including increased health-care costs and potential productivity losses at the population level.”

Lines 97-102:

“Because maternal nutritional status has been associated with long-term health outcomes in both women and offspring, women of reproductive age represents a relevant population in which to examine the prevalence and characteristics of DBM. [12]”

2. The authors state that the laboratory subsample (n = 5,212) was designed to be nationally representative and representative by area of residence. However, given the logistical requirements for biosample collection there is a potential risk of selection bias or non-participation bias compared to the full ENSPA-2019 survey sample (n = 17,997).

To strengthen the validity of the findings regarding the double burden of malnutrition, it would be beneficial for the authors to include a comparative analysis of the sociodemographic characteristics (e.g., age, education level, wealth quintile, and geographic region) between the full survey sample and the laboratory subsample of NpWRA.

Specifically:

Table Comparison: Could the authors provide a table (as an appendix or within the results) comparing these key characteristics to demonstrate that no significant systematic differences exist?

Response: The authors thanks to the reviewer for this in-depth analysis related to the validity and the potential selection bias. We acknowledge the concern regarding the potential selection bias arising from the limited sample size of NpWRA included in our analysis. To address this concern, we originally presented in Supplementary table #2 the baseline characteristics of NpWRA from the laboratory subsample included in this study and those from the same laboratory subsample who were excluded due to missing anthropometric/haemoglobin data. Nevertheless, to provide a more comprehensive assessment of the potential selection bias, we revised the Supplementary table #2 and now present comparisons (with their respective p-value for their chi2 test) by age group, area of residence, schooling, and family income, between the study sample and the total population of NpWRA from the ENSPA study.

Therefore, we added the following paragraph to lines 285-293 of the results section in the manuscript with track changes:

“S2 Table presents a comparison of women included and excluded from the analysis by age group, area of residence, schooling, and monthly family income. Overall, no statistically significant differences were observed between included and excluded women with respect to age, schooling, or family income. Although a statistically significant difference was identified for area of residence, differences across residence categories were smaller than 4%.”

3. Bias Discussion: If differences are found, please discuss how these might impact the prevalence estimates and how the complex sampling weights used in the analysis account for this potential bias.

This comparison would provide greater confidence that the subsample effectively preserves the representativeness of the original survey. If found that there is an important difference in distribution weights might need to be recalculated.

Response: Thank you for this observation. As recommended, we added a new paragraph discussing the results of the comparison between included and excluded women to the Limitations section (lines 577-583) of the manuscript with track changes, as follows:

“Finally, after applying the inclusion and exclusion criteria for the present analysis, the final sample size of NpWRA was smaller than the total ENSPA sample, raising the possibility of selection bias and potentially affecting the representativeness of the findings. Nevertheless, comparisons between women included and excluded from the analysis showed no statistically significant differences in age, education level, or family income. Although a statistically significant difference was observed for area of residence, the magnitude of this difference was small.”

4. The data collection section is very comprehensive and provides a clear understanding of the study's execution. However, as the authors note that this methodology has been described in detail elsewhere (e.g., in the primary ENSPA-2019 report or previous publications), the current description in the manuscript is quite lengthy.

To improve the flow and readability of the publication, I suggest the following:

Summarize the Core Methods: Keep only the essential information necessary for the reader to understand the current analysis (e.g., the specific variables used for the double burden of malnutrition and the laboratory subsample flow).

Move Detailed Protocols to an Annex: Transfer the more granular procedural details—such as specific equipment brands, detailed field staff training protocols, and step-by-step biosample handling—to a Supplementary Materials or Annex section.

Cross-Reference: Ensure that the text clearly cites the original methodological publication or the new Annex for readers who require the full technical details.

This adjustment would allow for a more concise manuscript that focuses more directly on the study’s specific findings regarding non-pregnant women of reproductive age.

Response: The authors thank for this suggestion. As recommended, we revised and summarize in the revised manuscript the core methods for the data collection of anthropometric variables and the step-by-step of the biosample handling. We now present the detailed procedures and the instruments used in a Supplementary 1 Appendix document.

5. The Discussion section (pages 31–40) provides a thorough comparison of the study’s findings with similar data from other countries. However, much of the current te

Attachment Submitted filename: Detailed_response_to_reviewers.docx

# Peer review: Decision Letter 1

**DOI:** 10.1371/journal.pone.0356784.r003

**Authors:** Guoying Wang

10 Aug 2026

Double burden of malnutrition among non-pregnant women of reproductive age: results from the National Health Survey of Panama (ENSPA) 2019.

PONE-D-26-04070R1

Dear Dr. Montenegro Mendoza,

We’re pleased to inform you that your manuscript has been judged scientifically suitable for publication and will be formally accepted for publication once it meets all outstanding technical requirements.

Within one week, you’ll receive an e-mail detailing the required amendments. When these have been addressed, you’ll receive a formal acceptance letter and your manuscript will be scheduled for publication.

An invoice will be generated when your article is formally accepted. Please note, if your institution has a publishing partnership with PLOS and your article meets the relevant criteria, all or part of your publication costs will be covered. Please make sure your user information is up-to-date by logging into Editorial Manager at Editorial Manager® and clicking the ‘Update My Information' link at the top of the page. For questions related to billing, please contact billing support.

If your institution or institutions have a press office, please notify them about your upcoming paper to help maximize its impact. If they’ll be preparing press materials, please inform our press team as soon as possible -- no later than 48 hours after receiving the formal acceptance. Your manuscript will remain under strict press embargo until 2 pm Eastern Time on the date of publication. For more information, please contact onepress@plos.org.

Kind regards,

Guoying Wang, MD, PhD

Academic Editor

PLOS One

Additional Editor Comments (optional):

Reviewers' comments:

Reviewer's Responses to Questions

**Comments to the Author**

Reviewer #1: All comments have been addressed

Reviewer #2: All comments have been addressed

**********

2. Is the manuscript technically sound, and do the data support the conclusions?

Reviewer #1: Yes

Reviewer #2: Yes

**********

3. Has the statistical analysis been performed appropriately and rigorously?

Reviewer #1: Yes

Reviewer #2: Yes

**********

4. Have the authors made all data underlying the findings in their manuscript fully available?

The PLOS Data policy

Reviewer #1: Yes

Reviewer #2: Yes

**********

5. Is the manuscript presented in an intelligible fashion and written in standard English?

Reviewer #1: Yes

Reviewer #2: Yes

**********

Reviewer #1: The authors have addressed all points in previous review. It addresses a very important topic and is well written and analyzed .It is fit for publication

Reviewer #2: Thank you for the opportunity to review the revised manuscript. I appreciate the authors' careful responses to the reviewers' comments and the revisions made throughout the manuscript.

The authors have addressed the majority of my previous concerns. In particular, the additional clarification of the methodology, discussion of missing data, and improvements to the data availability statement have strengthened the manuscript. The revisions have improved the clarity and transparency of the study.

**********

what does this mean?). If published, this will include your full peer review and any attached files.

If you choose “no”, your identity will remain anonymous but your review may still be made public.

**Do you want your identity to be public for this peer review?**  For information about this choice, including consent withdrawal, please see our Privacy Policy

Reviewer #1: No

Reviewer #2: No

**********

# Editor report: Acceptance letter

**DOI:** 10.1371/journal.pone.0356784.r004

**Authors:** Guoying Wang

PONE-D-26-04070R1

PLOS One

Dear Dr. Mendoza,

I'm pleased to inform you that your manuscript has been deemed suitable for publication in PLOS One. Congratulations! Your manuscript is now being handed over to our production team.

At this stage, our production department will prepare your paper for publication. This includes ensuring the following:

* All references, tables, and figures are properly cited

* All relevant supporting information is included in the manuscript submission,

* There are no issues that prevent the paper from being properly typeset

You will receive further instructions from the production team, including instructions on how to review your proof when it is ready. Please keep in mind that we are working through a large volume of accepted articles, so please give us a few days to review your paper and let you know the next and final steps.

Lastly, if your institution or institutions have a press office, please let them know about your upcoming paper now to help maximize its impact. If they'll be preparing press materials, please inform our press team within the next 48 hours. Your manuscript will remain under strict press embargo until 2 pm Eastern Time on the date of publication. For more information, please contact onepress@plos.org.

You will receive an invoice from PLOS for your publication fee after your manuscript has reached the completed accept phase. If you receive an email requesting payment before acceptance or for any other service, this may be a phishing scheme. Learn how to identify phishing emails and protect your accounts at https://explore.plos.org/phishing.

If we can help with anything else, please email us at customercare@plos.org.

Thank you for submitting your work to PLOS One and supporting open access.

Kind regards,

PLOS ONE Editorial Office Staff

on behalf of

Dr. Guoying Wang

Academic Editor

PLOS One
